# Toward Practical High‐Energy and High‐Power Lithium Battery Anodes: Present and Future

**DOI:** 10.1002/advs.202105213

**Published:** 2022-01-31

**Authors:** Caoyu Wang, Chunpeng Yang, Zijian Zheng

**Affiliations:** ^1^ Hubei Collaborative Innovation Center for Advanced Organic Chemical Materials Key Laboratory for the Green Preparation and Application of Functional Materials Ministry of Education Hubei Key Laboratory of Polymer Materials School of Materials Science and Engineering Hubei University Wuhan 430062 P. R. China; ^2^ School of Chemical Engineering and Technology Tianjin University Tianjin 300072 P. R. China

**Keywords:** high energy, Li anodes, Li batteries, P anodes, Si anodes

## Abstract

Lithium batteries are key components of portable devices and electric vehicles due to their high energy density and long cycle life. To meet the increasing requirements of electric devices, however, energy density of Li batteries needs to be further improved. Anode materials, as a key component of the Li batteries, have a remarkable effect on the increase of the overall energy density. At present, various anode materials including Li anodes, high‐capacity alloy‐type anode materials, phosphorus‐based anodes, and silicon anodes have shown great potential for Li batteries. Composite‐structure anode materials will be further developed to cater to the growing demands for electrochemical storage devices with high‐energy‐density and high‐power‐density. In this review, the latest progress in the development of high‐energy Li batteries focusing on high‐energy‐capacity anode materials has been summarized in detail. In addition, the challenges for the rational design of current Li battery anodes and the future trends are also presented.

## Graphical abstract

The increasing development of battery‐powered vehicles for exceeding 500 km endurance has stimulated the exploration of lithium‐ion batteries with high‐energy‐density and high‐power‐density. In this review, proximate developments in various types of high specific energy lithium‐ion batteries are screened, focusing on silicon‐based anode, phosphorus‐based anode, lithium metal anode, and hybrid anode systems.

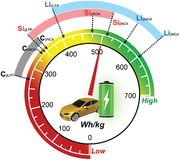

## 1 Introduction

Owing to their high energy density and long cycling life, rechargeable lithium‐ion batteries (LIBs) emerge as the most promising electrochemical energy storage devices beyond conventional lead‐acid, nickel‐iron, and nickel‐metal hydride.^[^ ^1^, ^2^ ^]^ Since the commercialization of LIBs in 1991, they have been quickly served as the main energy source for the smartphones, laptop computers, scooters, and electric vehicles required by today's information‐rich, mobile society.^[^ ^3^, ^4^ ^]^ The recent rise of the demand for high rate, high capacity, quick‐charging LIBs to meet the portable devices with prolonging stand‐by time, electric vehicles with long‐distance driving range (>500 km), and batteries with short charging time (<20 min), has stimulated research efforts in battery systems with high‐energy‐density and high‐power‐density.^[^ ^5^, ^6^ ^]^ However, LIBs based on Li‐ion intercalation electrode materials provide only limited energy density.^[^ ^7^ ^]^ One key issue for the increase of the overall energy density of the batteries is the anodes. To realize the goal of high energy density, three critical requirements must be met by the anode materials: i) a high Li storage capacity ensuring a high gravimetric/volumetric energy density; ii) a low standard redox potential of anode material enabling a high cell voltage; and iii) superior electron/Li^+^ conductivity facilitating a high‐rate capability.

The evolution of anode materials involved several stages including Li metal, high‐capacity alloy‐type anode materials, high‐capacity conversion‐type anode materials, and carbonaceous anode materials.^[^ ^8^ ^]^ In the 1970s, Li metal was firstly paired with layered‐type structural TiS_2_ cathode to assemble as rechargeable Li cells due to its high specific capacity (3860 mA h g^−1^) and low redox potential (−3.04 V vs standard hydrogen electrode).^[^ ^9^ ^]^ However, its practical application was soon hindered by the explosion of cells originating from dendritic Li growth. Alloy‐type anode materials with relatively high working potentials have been considered to address the dendritic Li obsession, but they usually demonstrated poor cycling stability related to the large volume expansion/contraction during charge/discharge cycles.^[^ ^10^ ^]^ Beyond alloys, high‐capacity conversion‐type materials including transition metal sulfides, oxides, and fluorides, were developed as a new class of advanced anode materials.^[^ ^11^ ^]^ The use of conversion‐type electrodes, however, was still restricted by a low energy density caused by their higher working potential (1.0−1.8 V vs Li^+^/Li), large voltage hysteresis, and high initial irreversible capacity.^[^ ^12^ ^]^ Later, a rechargeable LIB was assembled based on the stable Li^+^ insertion carbonaceous anodes in combination with transition‐metal oxides cathodes.^[^ ^13^ ^]^ Owing to the low equilibrium potential (0.1 V vs Li^+^/Li), low volume changes of less than 10%, high Li^+^/e^−^ conductivity, and long cycling life, graphite anodes enabled the wide application of LIBs in today's high‐performance portable electronic devices, electric vehicles, and hybrid electric vehicles.^[^ ^14^ ^]^ Practically, the energy densities of 300 Wh kg^−1^ have been achieved for power batteries. And the energy density of 730−750 Wh L^−1^ have been realized for “3C devices” (computer, communication, and consumer electronics).^[^ ^15^ ^]^ However, due to the occupation of Li between two adjacent graphene planes, the maximum composition of lithium intercalated graphite (LiC_6_) can be achieved to yield a useable specific capacity of 372 mA h g^−1^.^[^ ^16^ ^]^ The high Li activities of lithium/carbon intercalation compounds and their poor thermodynamically stability in all known electrolytes pose the decomposition of electrolytes to form an ionically conducting but electronically insulating “solid electrolyte interphase” (SEI) film during the first few Li^+^ intercalation/deintercalation cycles. SEI formation and corrosion‐like reactions of Li*_x_* C_6_ irreversibly consume both lithium and electrolyte, leading to large irreversible specific charge and low initial Coulombic efficiency (CE).^[^ ^17^ ^]^ Moreover, the lithiation and delithiation are associated with low equilibrium potential, approaching the depositing potential of metallic Li, which may cause the formation of dendrite upon cycling, giving rise to serious security problems.^[^ ^18^, ^19^, ^20^ ^]^

Considering the battery's safety and long cyclic life, LIB anodes experienced significant revolution from Li metal to graphite.^[^ ^21^ ^]^ In order to cater for energy density demands of various devices, from small consumer electronics to large vehicles, governments and institutions have been strongly supported and set targets for high energy batteries.^[^ ^22^ ^]^ For instance, the US Department of Energy (DOE) launched a “Battery 500 Consortium” to reach 500 Wh kg^−1^ battery energy density; New Energy and Industrial Technology Development Organization (NEDO) of Japan also released “Research and Development Initiative for Scientific Innovation of New Generation Battery” (RISING II) project to realize 500 Wh kg^−1^ in 2030; Chinese government established “Made in China 2025” project, targeting to stepwise reach 400 Wh kg^−1^ in 2025. However, energy densities of LIB show a slow increase rate of less than 3% in the last 30 years, and it is difficult to achieve these targets following the current tendency of LIB.^[^ ^15^, ^23^ ^]^ Therefore, in the future, the growing demands for high energy density will boost the development of anode materials from low‐capacity graphite to high‐capacity silicon (Si), phosphorus (P), and Li. When commercial graphite, Si, and Li anodes are used, high‐voltage LiNi_0.8_Co_0.1_Mn_0.1_O_2_ (NCM811, ≈200 mA h g^−1^) cathode‐based batteries provide gravimetric energy densities of 338, 473, and 555 W h kg^−1^, respectively.^[^ ^11^ ^]^ Replacement of the graphite by Si, P, and Li gives rise to a significant jump in both gravimetric and volumetric energy density.^[^ ^24^ ^]^ Furthermore, composite‐structure anode materials, such as Si/graphite, P/graphite, and Li/graphite, that inherit from the advantages of the single electrode are encouraged to improve both the capacity and stability of LIB anodes.

**Figure 1:**
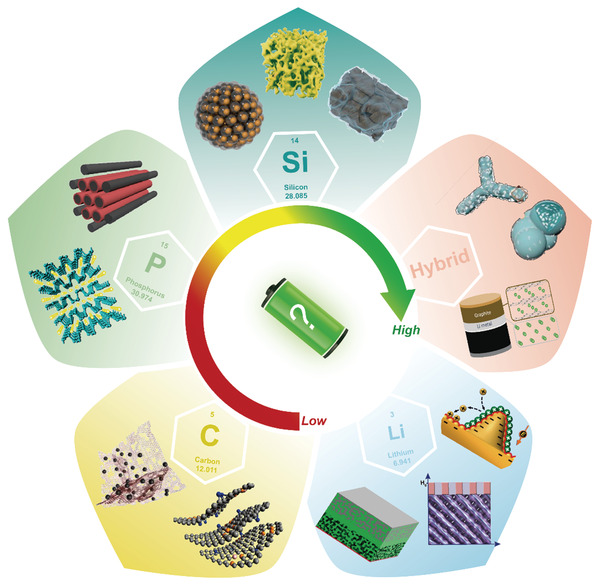
Ideal Li battery anodes for achieving high gravimetric energy densities. Images clockwise starting from the top image: Reproduced with permission.^[^ ^31^ ^]^ Copyright 2019, Springer Nature. Reproduced with permission.^[^ ^28^ ^]^ Copyright 2017, AAAS. Reproduced with permission.^[^ ^36^ ^]^ Copyright 2017, American Chemical Society. Reproduced with permission.^[^ ^36^ ^]^ Copyright 2017, American Chemical Society. Reproduced with permission.^[^ ^33^ ^]^ Copyright 2020, Elsevier. Reproduced with permission.^[^ ^34^ ^]^ Copyright 2013, American Chemical Society. Reproduced with permission.^[^ ^35^ ^]^ Copyright 2019, Elsevier. Reproduced with permission.^[^ ^32^ ^]^ Copyright 2018, National Academy of Sciences. Reproduced with permission.^[^ ^25^ ^]^ Copyright 2010, American Chemical Society. Reproduced with permission.^[^ ^29^ ^]^ Copyright 2019, The Royal Society of Chemistry. Reproduced with permission.^[^ ^26^ ^]^ Copyright 2012, American Chemical Society. Reproduced with permission.^[^ ^27^ ^]^ Copyright 2016, American Chemical Society. Reproduced with permission.^[^ ^30^ ^]^ Copyright 2014, Springer Nature.

In this review, we focus on the recent advance in high‐capacity, high‐rate, and low‐voltage electrode materials including Si, P, Li, and their composites used in the lithium battery anodes (**Figure** **1**). All these anode materials with merits and demerits are discussed in detail. Perspectives and suggestions for both current and future anode materials to enhance the energy density, rate capability, and safety of LIBs are also presented.

## 2 Key Performance Measures

### 2.1 Energy Density

The LIB's energy density can be interpreted as the integral of its specific capacity in regards to its voltage, which is largely determined by the specific capacities and operating voltages for two electrode materials.^[^ ^37^ ^]^ The specific capacity reflects the electric energy stored or delivered in an electrode material, which can be expressed by the following equation

(1) Cap=nF/3.6M

where *Cap* is the specific capacity of electrodes, in unit of mA h g^−1^; *M* is the molecular weight of the active material; *n* refers to the number of electrons transfer per formula unit of reactant, which can be decimal; F is the Faraday constant. According to this equation, the specific capacity is related to the electrons transfer number as well as the molecular weight of reactant, hence, electrode materials with more electrons transfer and lower molecular weight are in favor of high specific capacity. The battery voltage is equal to the potential difference between the cathode and the anode. Therefore, cathode materials with high‐capacity and high‐voltage as well as anode materials with high‐capacity and low‐voltage have been developed to improve the energy densities of LIBs. This review will mainly focus on the anode materials. C, P, Si, and Li delivers a theoretical specific capacity of 372, 2596, 3579, and 3861 mA h g^−1^ corresponding to an average voltage of 0.17, 0.8, 0.4, and 0.0 V, respectively, which has been considered as the present and future most promising anodes and will be discussed in detail below.

### 2.2 Power Density

High power applications such EV market demands drive the exploration of high rate and quick‐charging LIBs. Quick‐charging requires electrode materials that can be charged to a maximum specific capacity at a high current density, which may lead to lower active material utilization, faster material degradation, poor cycling performance, and severe heat release.^[^ ^38^ ^]^ Most electrode materials commonly exhibit poor high‐rate capability due to their intrinsically poor electron conductivity and sluggish Li^+^ diffusion. Kinetic improvements in terms of electrochemical kinetics and transport kinetics have been proven effective in achieving high‐rate performance. Improving the intrinsic electronic conductivity of the electrodes or introducing conducting networks into the electrodes facilitates rapid electron transport. Shortening Li^+^ ions diffusion or transport pathway via designing hierarchical open porous structures or downsizing the electrode materials allow fast Li^+^ ions transfer. Electrodes with enhanced kinetic performance are expected to achieve high‐rate performance with a high charge‐discharge current density of >5 A g^−1^.

## 3 Anode Materials

### 3.1 Silicon‐Based Anode Materials

**Figure 2:**
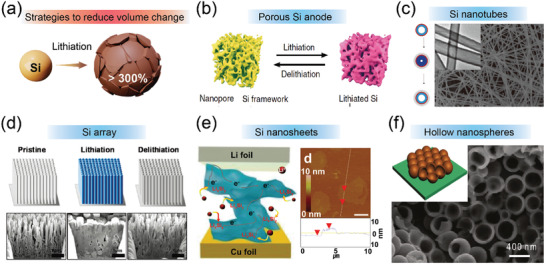
a) Electrode pulverization of Si. b) Schematic of porous Si anode. Reproduced with permission.^[^ ^31^ ^]^ Copyright 2019, Nature Publishing Group. c) SEM image of the Si nanotubes, inset the TEM image indicates the hollow nature of the sample. Reproduced with permission.^[^ ^47^ ^]^ Copyright 2012, Nature Publishing Group. d) Schematic illustration and cross‐sectional SEM images of Si array. Reproduced with permission.^[^ ^48^ ^]^ Copyright 2010, American Chemical Society. e) Schematic and AFM images of Si nanosheets. Reproduced with permission.^[^ ^49^ ^]^ Copyright 2016, American Chemical Society. f) Schematic and SEM image of hollow Si nanospheres. Reproduced with permission.^[^ ^50^ ^]^ Copyright 2011, American Chemical Society.

Because silicon (Si) has a high theoretical lithiated capacity (4200 mA h g^−1^) and Si is abundant resources, low cost, and environmental benign, numerous researches have been focused on Si‐based anodes.^[^ ^39^ ^]^ Moreover, Si has an average lithiation voltage of 0.2 V, which is slightly higher than that of graphite of 0.1 V, making it has higher safety. Even so, its practical application is restricted by its poor cycling stability arising from the low electronic conductivity, and low diffusion coefficient of Li^+^, and huge volume change of Si during alloying/dealloying processes.^[^ ^40^ ^]^ The low electronic conductivity of Si of 10^−3^ S cm^−1^ together with the low diffusion coefficient of Li^+^ (10^−14^ to 10^−13^ cm^2^ s^−1^) in Si materials, gives rise to poor kinetics and low utilization of its full capacity.^[^ ^41^ ^]^ Phase transformation and the accumulation of pulverized Si particles are two main factors for severe volume change.^[^ ^42^ ^]^ It is reported that each silicon atom can accommodate 4.4 lithium atoms to generate the fully lithiated state of the Li_22_Si_5_ phase, leading to about 400% volume expansion.^[^ ^43^ ^]^ Wherein, the transformation from elemental Si phase to lithiated Li_22_Si_5_ phase accounts for more than 300% increase in volume change (**Figure** **2**a).^[^ ^44^ ^]^ Induced by the anisotropic volume changes of Si anodes, large internal stresses are generated and applied to Si particles, leading to the cracking and pulverization of the Si‐based anode. The pulverized Si particles may be detached from the conductive materials or current collector, forming inactive components and porous structures. The continuous accumulation of inactive Si particles also contributes to the volume expansion of Si‐based anodes. Moreover, due to the loss of active Si, the anode suffers from rapid capacity fade, low utilization of Si, and short cyclic life. In addition, the large volume expansion and shrinkage of Si anode cause the constant breaking and re‐forming of the SEI film, causing a low Coulombic efficiency and poor rate capability.

To address the abovementioned issues with respect to Si, numerous efforts including designing nanostructured Si anodes, incorporating pores or voids inside the Si anodes, creating protective layers on Si anodes, and fabricating composite Si anodes, have been employed to improve the electrochemical performance of Si‐based anodes.^[^ ^45^ ^]^ Downsizing the Si particle size to micro/nanoscale, such as nanoparticles (NPs), porous Si, 1D material like nanowires and nanotubes, hollow NPs, 2D film‐like Si and Si nanosheets, and 3D Si structures, has been regarded as the most effective approach to improve the reversibility and electrochemical activity of Si (Figure 2b–f).^[^ ^46^ ^]^ Compared with the bulk Si counterparts, nanostructured Si usually exhibits remarkably shortened transport pathway for both the Li ions and electrons, ensuring favorable rate capability. In addition, nanostructured Si produces relatively small internal stress during the Li‐ion insertion and extraction reactions, guaranteeing stable volume change and high capacities.

**Figure 3:**
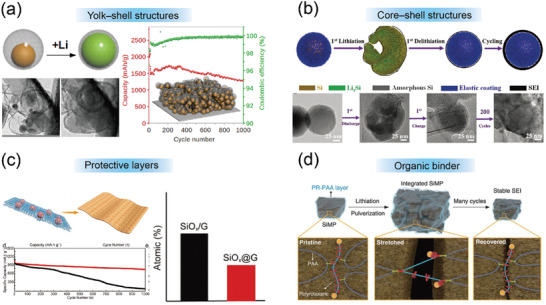
a) A magnified schematic and TEM images of Si@void@C particles, and its delithiation capacity and CE of the first 1000 galvanostatic cycles. Reproduced with permission.^[^ ^55^ ^]^ Copyright 2012, American Chemical Society. b) Schematic illustration showing the structural maintenance of the Si@a‐TiO_2_ nanoparticle electrode during lithium insertion and extraction. Reproduced with permission.^[^ ^45^ ^]^ Copyright 2017, Wiley‐VCH. c) Schematic and cycling performance of SiO*_x_* @G, together with its atomic percentage of F in different anodes after 1000 cycles. Reproduced with permission.^[^ ^58^ ^]^ Copyright 2018, Wiley‐VCH. d) Graphical representation of the operation of PR‐PAA binder to dissipate the stress during repeated volume changes of SiMPs. Reproduced with permission.^[^ ^28^ ^]^ Copyright 2017, American Association for the Advancement of Science.

Si nanoparticles with nanoscale diameters can realize quick relaxation of stress and thus resist mechanical fracture. The critical particle size is estimated to be around 150 nm, wherein, Si nanoparticles with a diameter of less than 150 nm can avoid fracture and pulverization while above which cracking occurs.^[^ ^51^ ^]^ However, for Si nanoparticle electrodes to be viable for practical batteries, two basic issues need to be addressed: i) Undesirable side reactions between Si and the electrolytes. Si nanoparticles usually have high specific surface area, which can promote the decomposition of electrolytes on the electrode surface. ii) Poor electrical contact between Si nanoparticles and the current collector as well as adjacent nanoparticles. Continual electrode expansion and contraction upon cycling gives rise to loss of electrical contact and finally contributes to poor reversibility and short cycle‐life. Si nanowires and nanotubes that are grown directly on the current collector can maintain robust electrical contact and provide rapid electronic pathway. However, the as‐prepared Si anodes still demonstrate rapid capacity fading due to large radial volume change (≈120%) and insufficient accommodation space for nanowires and nanotubes.^[^ ^52^ ^]^ For example, Cui and co‐workers grew Si nanowires on stainless steel substrates via a vapor–liquid–solid (VLS) method as the self‐standing Si anode.^[^ ^53^ ^]^ Although the as‐prepared Si anode exhibited a high reversible capacity of 3000 mA h g^−1^ and superior capacity retention with little capacity fading, only 10 cycles at 0.05 C were reported for the Si anode. Paik and co‐workers fabricated sealed Si nanotube arrays as the anode for LIB and found the as‐obtained anode encountered a 400% volumetric increase in radial direction but only relatively small (<35%) changes in the axial dimension (Figure 2d).^[^ ^48^ ^]^ Li and co‐workers investigated the anisotropic expansion of Si nanotubes via experiments and simulations, discovering the expansion rate of inner holes was less than the outside.^[^ ^54^ ^]^ They further verified that the fracture ratio is related to wall thickness and inner radius of Si nanotubes, and the optimal thickness–outer radius ratio is about 2/3. Under the optimal thickness–outer radius ratio, the outer diameter corresponding to a 0% fracture ratio is about 700 nm, which is over two times the critical diameter of Si nanowires, demonstrating the better fracture resistance of Si nanotubes. Si films have demonstrated high capacities and long cycle‐life because of the low active material loading, but rapid capacity fading caused by the cracking of films due to their anisotropic volume changes restricts their large‐scale application.^[^ ^41^ ^]^ Porous Si anodes including hollow spheres, hollow nanotubes, mesoporous Si sponge, micrometer‐sized Si nanoparticles, macroporous Si, yolk‐shell, and core‐shell structured Si‐based composites are also explored to improve the electrochemical performance of Si anode (**Figure** **3**a,b).^[^ ^45^, ^55^ ^]^ These nanostructured Si‐based anodes with porous structures can provide sufficient space or voids for lithiated Li_22_Si_5_ phase and withstand the internal stress induced by the anisotropic volume changes of Si anodes without cracking and pulverization, enabling the integration of Si anode. In addition, the abundant internal space and high surface area are in favor of the accessibility of electrolyte, ensuring a high‐rate capability. For instance, Cui et al. reported a Si nanotubes anode with impressive electrochemical performances regarding high reversible capacity, outstanding capacity retention, and favorable rate capability up to 5 C.^[^ ^47^ ^]^ Subsequently, sealed Si nanotube arrays were developed to accommodate the large volume changes of Si anodes, achieving high initial CE and stable capacity retention.

Despite improvements in the nanostructured Si anodes, which resist fracture, the poor interfacial stability resulting from their continuous volume expansion and contraction should not be ignored. The repeated formation and rupture of SEI film will pose the consumption of electrolyte and Li ions, prolong the Li^+^ diffusion distance, and insulate the electrical contact between the current collector and the Si nanoparticles and as well as adjacent nanoparticles. Therefore, designing a stable and strong SEI is crucial to achieving a long cycle life of Si anodes. The substantial volume change of Si electrode is mainly caused by the phase transformation and the accumulation of pulverized Si particles; wherein, the volume expansion resulting from the phase transformation is considered inevitable, but the cracks and inactive Si particles generated during volume expansion is controllable. Hence, the key to preparing a stable nanostructured Si anode is to alleviate the stress and maintain the electrode integrity. Decorating the Si anodes with various protective layers has been proven effective in stabilizing the anode/electrolyte interface, maintaining electrical connection, and buffering volume expansion. Several critical characteristics regarding the protective layers should be considered. They should have: i) high conductivity to enable fast Li^+^ transport; ii) suitable mechanical modulus to buffer or disperse the stress caused by the huge volume change; iii) high electrochemical stability to protect silicon electrode from the erosion by the electrolyte. To the best of our knowledge, the coating materials are mainly composed of inorganic layers and organic layers. Inorganic layers include various metals, metallic‐/nonmetallic oxides, carbon‐based materials, carbon nanotube, and graphene, which have excellent conductivity, high‐chemical and thermal stability, and superior mechanical flexibility. Li et al. explored the thickness effect of the SEI film on the electrochemical performance of Si nanocone anode by SEM characterization.^[^ ^56^ ^]^ The Si anode with thin SEI layer demonstrated higher rate capability than that of the anode with thick SEI film. Graphene‐coated Si nanocone enabled a thin SEI layer formed on its surface and the resultant anode delivered a long cycle life of 1715 cycles with high Coulombic efficiency of 98.2%.^[^ ^57^ ^]^ Subsequently, Guo et al. proposed a facile fabrication approach to address disadvantages of SiO*_x_*‐containing graphite, such as low tap density, huge volume variation, and poor electronic conductivity (Figure 3c).^[^ ^58^ ^]^ Organic layers refer to conducting polymer‐based layers, such as polyaniline (PANi), polypyrrole (PPy), self‐healing polymers (SHPs), and poly(3,4‐ethylenedioxythiophene)/poly(styrene‐4‐sulfonate) (PEDOT:PSS) that can reinforce the SEI layer, enhance the structural stability, and maintain the electrical and mechanical integrity of the Si‐based electrodes.^[^ ^59^, ^60^, ^61^ ^]^ The nanostructured Si and various protective layers can alleviate the volume expansion to a certain extent, at the same time, binder with high mechanical properties is also essential to achieve long‐term cycle life.

In addition, reinforcing the Si anode with in situ SEI film formed via optimizing electrolyte components and introducing electrolyte additives is proven effective in suppressing capacity attenuation.^[^ ^62^ ^]^ For instance, FEC‐originated SEI that is denser and thinner than that of porous and permeable films formed in FEC‐free electrolytes tends to protect against the decomposition of electrolyte and the oxidation of Si anode.^[^ ^63^ ^]^ VC additive is considered to enhance the compactness and stability of the SEI film, which outperformed other additives such as FEC, LiBOB, and VEC at same composition.^[^ ^64^ ^]^ Apart from these additives, other types of additives, such as nitrogen‐containing additives (such as cyanate, isocyanate, thiocyanate, and isocyano, etc.), silane‐type additives, carbon dioxide (CO_2_), and ionic additives (such as LiBOB, LiFOB, and LiDFOB, etc.), are also employed in Si anodes to improve the SEI film, and to achieve prolonged cycle lifespan.^[^ ^65^ ^]^ Considering the economic benefits, additives featuring low‐cost, ecofriendly, effectiveness and durability will be welcomed in the near future. As a necessary component of the battery, binders play an important role in electrode preparation and electrochemical performance exerting. Fundamentally, the following characteristics should be considered in designing novel binders for Si anode: i) good dispersion; ii) mechanical integrity; iii) weak interaction with the electrolyte; (iv) high viscosity. Based on these principles, natural‐originated binder obtained from animals or plants (such as carboxyl methylcellulose (CMC), guar arabic (GA), guarm gum (GG)), and synthetic binders (such as polyacrylic acid (PAA), polyvinylidene fluoride (PVDF), polyacrylonitrile (PAN), etc.) have been developed as Si anode binders. However, traditional binders are inadequate to resist enormous volume changes of Si due to poor interfacial interaction between anode and binder and limited toughness. Hence, decorating the existed binders with reactive functional groups such as –COOR/–COOH, –OH, and –NH_2_ is required.^[^ ^60^ ^]^ Besides, designing ultrahigh elastic binders with dynamical crosslink sites is urgent and important. In this text, Choi et al. proposed a highly elastic binder polyrotaxane. The incorporation of polyrotaxane (5 weight % of the binder portion) altered the mechanical properties of traditional binders such as PAA and kept pulverized particles together without disintegration during long‐term cycling (Figure 3d).^[^ ^28^ ^]^

Creating a composite structure that integrates a host matrix with Si nanoparticles is another feasible approach to improve the electrochemical performance of Si anode. First, the matrixes with high surface area act as dispersants to realize an even distribution of Si nanoparticles on its surface without agglomeration. Second, the matrixes with high conducting network enable a continuous electron pathway to provide better electrical contacts. Third, their high mechanical strength and favourable flexibility help buffer the stress and accommodate the volume change. Various inactive host matrixes, such as metal nitrides, metal carbides, and metal oxides were mixed homogenously with Si by ball milling to improve the structural stability of composite Si anodes.^[^ ^43^ ^]^ Apart from metallic compounds, metals including Fe, Ni, Cu, Mg, Ag, and Sn that can react with Si to form alloy anodes, were developed for Si dispersion due to their excellent electronic conductivity and good mechanical properties.^[^ ^66^ ^]^ Fe, Ni, and Cu in the elemental state in situ formed during the initial discharging/charging process and then homogeneously distributed in the composite as buffering matrix to alleviate Si volume variation.^[^ ^67^ ^]^ However, the poor interaction between metal and Si caused the segregation of metal from the Si surface due to substantial volume change of the Si particles upon cycling. Si‐based Mg, Ag, and Sn alloys that can reversible with Li^+^ by lithiation/delithiation processes show strong binding with Si particles, but mechanical cracking resulting from the complex alloying/dealloying reaction was detected. Due to its intrinsic merits of high structure stability, good physical and chemical stability, favorable electronic conductivity, abundant natural resources, nontoxic, and low cost, carbonaceous materials, especially graphite, were used for preparing Si/C composite anodes.^[^ ^68^ ^]^ In addition, conductive polymers were used as flexible buffer medium to alleviate large volume change.

In all, the nanostructured silicon anodes improve the cycling performance by reducing the dimensionality and shortening the transport pathway, but their high surface area can induce uncontrolled reaction and thermal runaway, causing large irreversible capacity loss and severe safety hazards. Protective layers show their potential to stabilize the Si/electrolyte interface, however, substantial research is required to ensure practical applications. The combination of a host matrix with Si nanoparticles is promising to improve the intrinsically slow dynamic of Li^+^ in Si anodes. However, the poor Li^+^ ion diffusivity in the inactive matrix and poor mechanical integrity of anodes usually lead to low reversible capacities and poor cycle life. Therefore, there is a need to introduce high electronic conductivity, favorable flexibility, and high stability surface coatings to maintain the intact electrical contact of Si anodes without pulverization.

### 3.2 Phosphorus‐Based Anode Materials

**Figure 4:**
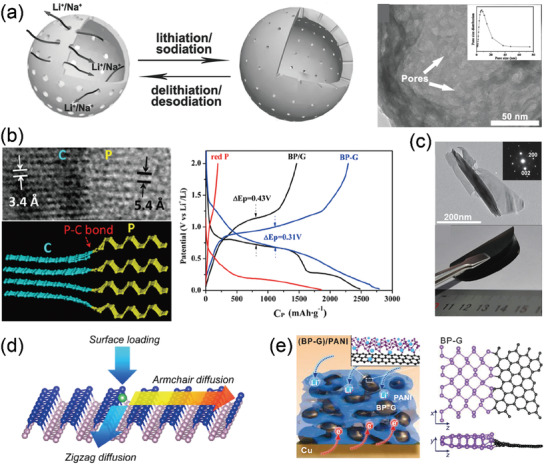
a) Schematic illustration of hollow nanospheres with porous shells during lithiation/sodiation and volume variation. Reproduced with permission.^[^ ^72^ ^]^ Copyright 2017, Wiley‐VCH. b) HRTEM image and schematic of BP‐G composite, together with the charge–discharge profiles of red P, BP/G, and BP‐G electrodes at the first cycle. Reproduced with permission.^[^ ^76^ ^]^ Copyright 2014, American Chemical Society. c) Schematic and cycling performance of SiO*_x_* @G, together with its atomic percentage of F in different anodes after 1000 cycles. Reproduced with permission.^[^ ^77^ ^]^ Copyright 2016, Wiley‐VCH. d) Schematic of Li surface loading (adsorption process), diffusion along armchair and zigzag directions on phosphorene surface. Reproduced with permission.^[^ ^78^ ^]^ Copyright 2015, American Chemical Society. e) Schematic of (BP‐G)/PANI, and BP‐G hybrid structure with in plane P–C bonds. Reproduced with permission.^[^ ^79^ ^]^ Copyright 2020, American Association for the Advancement of Science.

Elemental phosphorus has been developed as a promising anode material due to the fact that three Li^+^ can be reversibly inserted into the phosphorus to form Li_3_P, delivering a high theoretical capacity of 2595 mA h g^−1^.^[^ ^69^ ^]^ Solid phosphorus has three main allotropes: white, red, and black. White phosphorus (WP) is volatile and chemically unstable, which is not suitable for anode materials; thus, red phosphorus (RP) and black phosphorus (BP) have been widely investigated as anode materials.^[^ ^70^ ^]^ RP is attractive due to its intrinsic advantages of chemically stable, natural abundant, environmentally benign, and low‐cost.^[^ ^27^, ^71^ ^]^ However, the bulk or micron‐sized RP anode suffers from rapid capacity fading, which is mainly attributed to its low electrical conductivity and large volume variation during cycling. Nanosized RP can significantly increase the reversible capacity, but the capacity fading still occurs due to the large volume change. Hollow nanospheres with porous shells are recognized as an ideal structure to resolve these issues. Wang et al. fabricated hollow P nanospheres (HPNs) with porous shells via a gas‐bubble‐directed formation mechanism (**Figure** **4**a). Due to the merits of the porous and hollow structures, the HPNs revealed a high capacity of 1285.7 mA h g^−1^ and excellent long‐cycling performance.^[^ ^72^ ^]^ Amorphousizing or constraining P in high conducting carbon scaffolds have proven effective in alleviating large electrode volume changes. For example, Yang et al. fabricated an amorphous P/C nanocomposite anode, which delivered a high reversible capacity of 2355 mA h g^−1^ based on 3‐Li storage reactions and maintained 90% of capacity over 100 cycles.^[^ ^73^ ^]^ Especially, a nanostructured P/C composite prepared by a vaporization/adsorption strategy showed a high P utilization of 90%.^[^ ^74^ ^]^

Similar to that of graphite, orthorhombic BP shows 2D layered structures, which is beneficial for lithium diffusion with a low Li^+^ diffusion barrier. The electrical conductivity of BP reaches ≈300 S m^−1^, which is significantly higher than that of RP of 1 × 10^−12^ S m^−1^, allowing for a high‐rate lithium storage. In addition, it has high thermodynamic and chemical stability, low band gap (0.34 eV), and reasonable density (2.69 g cm^−3^), making it a high‐capacity anode material. The seminal work of developing the BP as anode material was conducted by Park and John in 2007.^[^ ^75^ ^]^ The BP anode prepared by high energy mechanical milling showed a high initial discharge and charge capacity, but it suffered from poor cycling performance with only a charge capacity of 220 mA h g^−1^ after 30 cycles. The underlying reason was mainly caused by its semiconductor feature and the significant volume change during cycling. A P/C composite anode that P was finely dispersed in the carbon scaffold was proven effective in improving its reversibility, Coulombic efficiency, and cycling stability of BP (Figure 4b).^[^ ^76^ ^]^ Moreover, the P‐C bonds formed via mechanochemical reaction can enable an intact contact between P and carbon matrix, thus affording high capacity and cycle stability. However, the BP electrode usually suffers from poor interfacial kinetics due to the edge atom reconstruction near the zigzag diffusion channel. Hence, few‐layer BP and phosphorene that have rapid Li^+^ mobility are developed as high rate anode materials (Figure 4c,d).^[^ ^77^, ^78^ ^]^ Recently, Ji et al. fabricated a (BP‐graphite)/polyaniline (PANI) composite anode by ball milling in combination with coating strategy for high‐rate high‐capacity lithium storage (Figure 4e).^[^ ^79^ ^]^ The BP can covalently bond with graphite flakes to form strong P–C bond, mitigating the edge reconstruction problem and affording Li^+^ transport. Whereas, the PANI coating could prevent the continuous formation of poorly conducting Li fluorides and carbonates, maintaining stable electrode–electrolyte interfacial morphology and structure. Benefiting from the synergistic effect of P–C bonds and PANI coating, the reversible capacities for the (BP‐graphite)/PANI anode reached 910, 790, and 440 mA h g^−1^ under the high current density of 2.6, 5.2, and 13 A g^−1^, respectively.

In a word, RP and BP emerge as potentially attractive anodes due to their high specific capacity. However, due to the insulating nature of RP, the RP anodes usually demonstrate poor electrochemical activity and cycling stability. Integrating RP and highly conducting carbon scaffold is effective in improving the intrinsically electrical conductivity and alleviating the volume change of RP anode, but the introduction of inactive carbon matrix may impair the composite capacity and cell's energy density. BP shows its superior in electronic conductivity and Li^+^ diffusion, however, the large‐scale preparation of high‐quality bulk BP with high efficiency remains a significant challenge. Atomically thin BP may be used as a high‐rate and high‐capacity anode due to its unique electronic property and structural advantage if one can realize a scalable and ecofriendly synthesis of layered BP.

### 3.3 Lithium Metal Anodes

**Figure 5:**
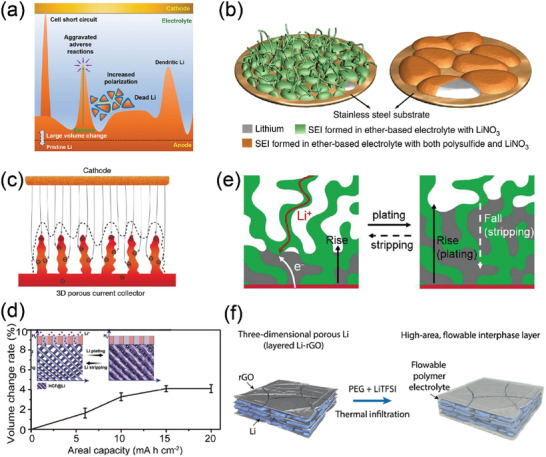
a) Dilemmas of Li‐metal Anodes. Reproduced with permission.^[^ ^80^ ^]^ Copyright 2017, American Chemical Society. b) Schematic illustration showing the morphology difference of lithium deposited on the stainless, with or without the polysulfide and LiNO_3_. Reproduced with permission.^[^ ^87^ ^]^ Copyright 2015, Nature Publishing Group. c) Illustration of the proposed electrochemical deposition processes of Li metal on 3D current collector. Reproduced with permission.^[^ ^93^ ^]^ Copyright 2015, Nature Publishing Group. d) Electrode volume change rate of MOF‐HCF@Li anode after plating, inset the schematic illustration of the Li plating/stripping process. Reproduced with permission.^[^ ^35^ ^]^ Copyright 2019, Elsevier. e) Schematic for the process of Li deposited in the 3D ion‐conductive host from the bottom current collector. Reproduced with permission.^[^ ^32^ ^]^ Copyright 2018, National Academy of Sciences. f) Schematics illustrating the fabrication process of the 3D Li anode with flowable interphase for solid‐state Li battery. Reproduced with permission.^[^ ^105^ ^]^ Copyright 2017, American Association for the Advancement of Science.

Li metal has been considered as the ultimate anode material due to its high theoretical capacity (3860 mA h g^−1^) and low redox potential (−3.04 V vs standard hydrogen electrode).^[^ ^80^, ^81^ ^]^ The energy densities of batteries pairing with the Li metal anode outperform those of batteries pairing with graphite anode and silicon anode. However, two critical issues regarding dendritic Li growth and unstable electrode–electrolyte interface restrict its practical application (**Figure** **5**a).^[^ ^82^ ^]^ Due to the high diffusion barrier and weaker interaction energy, Li atoms tend to grow into 1D long tubular‐shaped or filament‐like Li dendrites.^[^ ^83^ ^]^ Li dendrites customarily have a high surface area, which can induce the parasitic reaction between Li metal and electrolyte, contributing to low Coulombic efficiency. The continuously growing Li dendrites pierce the separator and reach the cathode, giving rise to the internal electrical contact and short‐circuit of a working cell. The short‐circuit arising from Li dendrites causes cell thermal runaway and fire/explosion risk. The prolonged Li dendrites may detach from the current collector and convert into electrochemically inert dead Li.^[^ ^84^ ^]^ The accumulation of dead Li accounts for low Coulombic efficiency, low Li utilization, and porous structure of Li anode.^[^ ^85^ ^]^ Another issue that needs special attention is the unstable interface. It's known that Li metal is thermodynamically unstable, and easily reacts with solvents or additives in the electrolyte, forming a thin and unstable solid electrolyte interphase (SEI) on the surface. During cycling, SEI's cracking leads to the continuous consumption of electrolytes and Li. In addition, due to the electrical insulation of the SEI layer, Li dendrites wrapped by SEI falling off from the electrode become “dead Li,” which results in a sharp decrease in the battery capacity and huge volume expansion.^[^ ^86^ ^]^

An ideal Li anode should be safe, featuring dendrite‐free, high Coulombic efficiency, and a long cycle lifespan. To achieve this goal, researchers have developed many strategies. Among them, rational electrolyte design is a simple and effective method to protect Li anode. By adding electrolyte additives, the in‐situ formed SEI layer can be more stable. For example, Cui and co‐workers added LiNO_3_ and lithium polysulfide to the electrolyte, which can lead to a uniform and stable SEI layer to prevent further reaction of Li and electrolyte (Figure 5b), and thus significantly improved the CE in a long cycling (≈99% for over 300 cycles).^[^ ^87^ ^]^ With the deepening research, a variety of electrolyte additives have been developed, such as FEC, CsPF_6_, LiPF_6_, LiNO_3_, AlCl_3_, LiTFSI, and LiFSI.^[^ ^34^, ^88^ ^]^ In the future, large voltage window, wide working temperature range, and less electrolyte addition (<3 g Ah^–1^) will be the research interests of electrolyte design.

The strategy of electrolyte additives can improve the deposition behavior of Li to a certain extent. However, in the application requiring high current density, excessive local current density will aggravate the growth of Li dendrite. In order to meet the practical needs of high current density, many studies have shown that high density of nucleating active sites is the key to the homogeneous growth of Li.^[^ ^89^, ^90^, ^91^ ^]^ Therefore, 3D current collectors, such as 3D porous Cu with high electroactive surface area, unstacked 3D graphene framework, 3D carbon nanofibers, etc., are considered to be one of the effective strategies to alleviate dendrite growth.^[^ ^92^ ^]^ Compared with the traditional planar current collector, the 3D current collector can disperse the local current density, provide more active sites to induce the homogeneous Li deposition, and accommodate Li into the skeletons without significant volume change. Guo's group reported a 3D Cu collector for the first time, in which Li was deposited on a submicron fiber copper host (Figure 5c).^[^ ^93^ ^]^ The result showed that compared with 2D Cu foil, the deposition of Li on 3D Cu collector has higher CE, less dendrite formation, and thus the Li‐3D Cu anode had a long cycle lifespan. In addition, porous 3D structures are demonstrated to accommodate the volume expansion of lithium and guide stable cycling performance. However, 3D current collectors usually show an undesirable affinity with Li metal, which may result in large nucleation overpotential and poor contact. To solve this problem, various strategies such as noble metals, Li‐rich composite alloys, functional organic coatings, and elemental additives, functional group modification, are used to improve the lithiophilicity.^[^ ^94^, ^95^ ^]^ In order to be more economical and convenient manufacture, Ye et al. developed a facile surface nanocrystallization strategy to promote a rapid wettability of molten Li.^[^ ^96^ ^]^ The metal nanoparticles randomly distributed on a 3D current collector decrease the surface energy and also gave rise to a Laplace pressure and thus enabled uniform Li deposition and low nucleation overpotential. Recently, the composite anode which integrates the advantages of SEI protective layer and 3D current collector has gradually become a research hotspot. Zheng and co‐workers further proposed the combination of 3D current collector and interface protective layer strategy to realize low volume change lithium metal anode (Figure 5d).^[^ ^35^ ^]^ By encapsulating Li into a hybrid host, a 3D conducting scaffold coated with metal‐organic frameworks (MOFs), MOFs layer with high Young's modulus boost uniform distribution of Li ions, and the thus‐formed anode displayed even Li deposition with a small dimension variation (<5%). At present, various kinds of 3D collectors have been developed in Li anode, such as metal‐based host and carbon‐based host, and so on.^[^ ^96^, ^97^ ^]^ Nevertheless, due to the internal and external consistency of 3D collector, Li may still deposit on the surface to form dendrites, resulting in local short circuit. In the future, more research will focus on how to induce Li deposition in the 3D collector or using lithiophilicity gradients to shift the favorable nucleation spots from the potentially unsafe anode/separator interface to the safe anode bottom. Another issue worthy of attention: high effective surface area of 3D current collector will lead to more SEI formation, and cause a severe consumption of Li and electrolyte during the cycling. Therefore, how to limit excessive SEI formation and clarify the effect of porosity on Li deposition need further research and optimization.

Traditional liquid electrolytes mostly use organic solvents, which are volatile and flammable.^[^ ^98^, ^99^ ^]^ Therefore, it is easy to pose a safety hazard. In recent years, the solid‐state electrolyte (SSE) has gradually become a hotspot because its feature of incombustible, nonvolatile, nonflammable, and stable at elevated temperatures.^[^ ^100^, ^101^ ^]^ For instance, Hu and co‐workers developed a host that is based on a garnet‐type ion‐conductive framework and a bottom deposited Cu current collector (Figure 5e).^[^ ^32^ ^]^ The Li anode was plated within the solid garnet framework from the bottom Cu layer and showed a dendrite‐free deposition behavior, effectively averting the dendrite penetration issue. In addition, in the extreme temperature environment where the liquid electrolyte cannot work, the solid electrolyte is still capable (−50 to 200 ℃). However, solid‐state electrolytes still face some challenges in practical applications: low ionic conductivity at room temperature and poor solid–solid interface contact are difficult to solve.^[^ ^102^ ^]^ It has been found that the reasonably designed composite electrolyte can effectively improve interface contact and increase ionic conductivity. For example, PEO‐based polymer is frequently introduced to composite inorganic ceramic (Li_7_P_3_S_11_‐PEO‐LiClO_4_, Li_6.4_La_3_Zr_1.4_Ta_0.6_O_12_/PEO/succinonitrile), for its proper flexibility can even the interface.^[^ ^103^ ^]^ What's more, in situ polymerization is also an important strategy to improve interface contact. In 2018, Guo et al. report that commercialized liquid electrolyte (DOL/DME) can be easily converted into a novel quasi‐solid gel polymer electrolyte via a simple in‐situ polymerization strategy.^[^ ^104^ ^]^ It is worth noting that SSE also can be combined with 3D current collectors to give free rein to their advantages. Cui and co‐workers adopted a 3D Li anode for the first time for the construction of solid Li batteries (Figure 5f).^[^ ^105^ ^]^ This structural design significantly increases the electrode–electrolyte contact area, dissipating the current density to facilitate charge transfer. More importantly, the incorporation of a flowable interfacial layer can accommodate the varying morphology at the 3D Li anode surface during cycling, which is desirable for maintaining continuous electrode–electrolyte contact. However, further research is still needed along with these directions such that good interfacial contact can be realized without compromising its nonflammability and mechanical properties. In the future, SSE design should be featured high mechanical strength, high ionic conductivity, and good interface contact as its main goals. Moreover, in order to improve the overall energy density of the battery, ultrathin solid electrolyte film is also one of the directions in future research.

Despite the progress in lithium anode stabilization, the excessive use of Li metal foils remains a critical yet unresolved challenge in this field. A practical LMB requires a thin Li metal foil with an areal capacity of less than 4 mA h cm^−2^ to pair with current cathodes (3 to 4 mA h cm^−2^).^[^ ^106^ ^]^ And due to its poor viscosity and mechanical processing performance, it is difficult to achieve the large‐scale preparation of ultrathin lithium metal foil by conventional mechanical rolling process.^[^ ^107^ ^]^ At present, micrometer‐thin Li foil is mainly fabricated through electrochemical deposition and vacuum evaporation methods, which are costly and show poor practicability. It is found that increasing the wettability of molten lithium is beneficial to large‐scale preparation of ultrathin lithium metal anode.^[^ ^108^ ^]^ A series of ultrathin Li metal anode has been prepared through the reaction of molten lithium with functional organic coatings or element additives.^[^ ^95^ ^]^ In addition, the introduction of ultrathin mechanically strong lithium host can significantly improve the mechanical strength of the micrometer‐thin Li foil and realize the free‐standing Li anode.^[^ ^106^ ^]^ Since the strategy is based on molten lithium, which consumes a lot of energy, it is essential to develop low energy consumption, low‐cost and simple technology for large‐scale fabrication of free‐standing ultrathin metallic Li anodes in future studies. In practical applications, the problem of Li consumption caused by “dead Li” will be more significant, so that the ultrathin lithium metal anode needs to achieve a higher coulombic efficiency to ensure the long‐term cycle life.^[^ ^109^ ^]^ In the future, the collaborative use of electrolyte additives, 3D current collectors, artificial SEI, solid‐state electrolyte, and other strategies in ultrathin lithium anodes will be an essential research direction.

In short, as the next‐generation high‐energy battery, Li metal anode has great commercial prospects in the field of portable battery equipment and new energy vehicles. Nonetheless, some problems are limiting the practical application of Li metal anodes, such as Li dendrites and unstable interfaces, which can cause serious volume expansion. The growth of Li dendrites can be inhibited by using strategies like reasonable electrolyte design, 3D current collector, and so on. Furthermore, the emerging SSE also provides a safer solution for Li metal anode. However, in high current density, Li metal anodes still face serious dendrite problems due to the high local current density normally accelerates the growth of Li dendrites and leads to an unstable cycling performance. In addition, as a nonrenewable resource, the use of excessive Li in production will cause a waste of Li resources. Therefore, the ultra‐thin lithium anode (<50 µm) with high Li utilization is the research direction in the future.

### 3.4 Silicon–Carbon and Lithium–Carbon Hybrid Anodes

The fast‐growing electric vehicle market increases its demands on advanced batteries with high energy density and low cost. In order to achieve popularization in the mass market, it is essential to develop a battery with a density of approximately 750 Wh L^−1^ with a corresponding driving range of more than 500 km (300 miles).^[^ ^110^ ^]^ However, it has been discussed above that it is difficult for LIBs to achieve such a high energy density on the basis of lithium insertion. Although batteries assembled with silicon anode and Li metal anode have much higher energy density than the graphite anode counterparts, their low cycle lifespan and poor safety have become the biggest obstacles for commercial use. Besides, in order to achieve a long cycle life, Li metal batteries often add excessive lithium and electrolyte to make up for the consumption during the cycling. All these have caused the cost of batteries too high to be used commercially.

**Figure 6:**
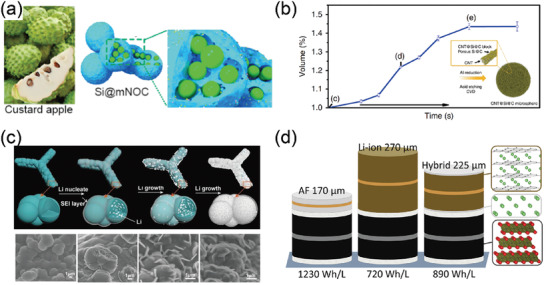
a) Schematic illustration of the hierarchical porous interconnected Si@N, O‐dual‐doped carbon. Reproduced with permission.^[^ ^113^ ^]^ Copyright 2017, Elsevier. b) Volume expansion curve of the particle recorded during the lithiation process, inset the schematic figure of CNT@Si@C microspheres. Reproduced with permission.^[^ ^111^ ^]^ Copyright 2020, Nature Publishing Group. c) Schematic diagrams showing the Li plating process on the CMN, and morphological evolution of Li plating on the CMN. Reproduced with permission.^[^ ^36^ ^]^ Copyright 2017, American Chemical Society. d) Schematic illustrating the thickness of the cell stack for anode‐free lithium metal, conventional lithium‐ion, and hybrid lithium‐ion/lithium metal cells based on the electrode loadings from the pouch cells. Reproduced with permission.^[^ ^33^ ^]^ Copyright 2020, Elsevier.

Before solving the problems of these anodes with high specific energy and realizing their commercial application, the value of hybrid anodes as a transitional solution cannot be underestimated. At present, neither graphite anode nor silicon anode can simultaneously meet two important indicators of modern electronic devices: high energy density and long lifespan. Therefore, it has been proposed to combine silicon with carbon materials which are used to improve the conductivity of the silicon anode and limit its volume expansion effect, thereby increasing the cycle life of the battery. At the same time, the doping of silicon can increase the overall energy density of the battery and realize the complementary advantages of the two materials. People have developed a variety of processes for preparing silicon–carbon composite materials, such as coated composite materials, embedded composite materials, doped composite materials, and so on.^[^ ^111^, ^112^ ^]^ For instance, Yu et al. developed a simple approach to rationally design and controllably synthesize custard apple‐like Si@N, O‐dual‐doped carbon with hierarchical porosity (**Figure** **6**a).^[^ ^113^ ^]^ Due to the merits of the hybrid structure design, it delivers outstanding reversible capacity at high current density with good rate capability and a long cycling life of over 4000 cycles as an anode for Li‐ion batteries. Furthermore, to inhibit the SEI excessive growth during repeated lithiation/delithiation, Qiu et al. designed a coaxial hollow nanocables of carbon nanotubes and silicon composite (CNTs@Silicon) structure.^[^ ^114^ ^]^ To address the poor mechanical strength of these porous structures, Zhang et al. designed hierarchical carbon‐nanotube@silicon@carbon microspheres to achieve both high porosity and extraordinary mechanical strength (>200 MPa) (Figure 6b).^[^ ^111^ ^]^ The hollow structured silicon tends to expand inward and shrink outward during lithiation/delithiation, and makes SEI stable. The favorable carbon doping in the hybrid anode can effectively buffer the volume effect of silicon, enhance the electronic conductivity, and produce a stable SEI film to maintain the interface between the composite material and the electrolyte.^[^ ^115^, ^116^ ^]^ However, in the traditional core–shell structured silicon–carbon composite material, during the lithium insertion process, the intense volumetric stress of silicon leads to the collapse of the hybrid anode's structure and the rapid decrease in capacity. Therefore, the future development direction of silicon–carbon composite anodes is preparing silicon and carbon materials with nanostructures and designing a reasonable composite structure to improve ion conduction inside the electrode and relieve stress changes during cycling.

At present, commercial silicon‐based anode materials are mainly composed of silicon oxide or nanosilicon and graphite respectively.^[^ ^117^ ^]^ By adding 5% to 10% of silicon material, the reversible capacity of silicon–carbon anode can reach 450 mA h g^–1^, which can partially meet application requirements of coulomb efficiency, cycle performance, and rate performance, and has begun to enter consumer electronics and electric vehicles in small batches. To further reach higher energy density silicon–carbon anodes, simply increasing the silicon content in the silicon carbon anodes will lead to problems such as low coulombic efficiency, large volume changes, and poor cycle stability.^[^ ^118^ ^]^ To take into account the energy density and cycle stability of the silicon–carbon anode, a lot of work in recent years has been around different dimensions of silicon (0D nanosilicon, 1D silicon nanotube/fiber, 2D silicon thin film, 3D bulk silicon), and many breakthroughs have been made in various indicators. The use of lower‐cost *μ*m‐scale silicon instead of nanoscale silicon has more commercial applications. Although they will mechanically break during cycling, the use of self‐healing polymer binders, new electrolytes, and strong graphene coatings can significantly improve the performance of micron particles.^[^ ^117^, ^119^ ^]^ In addition, different types of carbonaceous additives, such as carbon nanotubes, reduced graphene oxide, and pyrolytic carbon derived from precursors such as pitch, sugar, and heteroatom polymers, have also improved the electrochemical performance of the silicon–carbon anode.^[^ ^119^, ^120^ ^]^ From the point of view of commercialization in the future, the use of carbonaceous additives with low‐cost, and easy to expand the preparation of silicon–carbon composite anodes, is very important.

The strategy of the hybrid anode is also quite effective in graphite and Li metal anode. In the silicon–carbon hybrid anode, the complex preparation of nanosized silicon increases the cost of the electrode. Furthermore, it is urgent to have a reasonable structural design to relieve the internal stress of the electrode by optimizing the interface contact between graphite and silicon. Different from the silicon–carbon hybrid anode, the graphite and Li hybrid anode is more convenient in preparation. In this structure of hybrid anode, Li is stored in a lithium/carbon compound as a backup source through the lithium intercalation process, which is used to offset the irreversible loss of lithium during the cycle. In most studies, in order to achieve a long cycle life, a superfluous amount of lithium is often used (an average value of 300%), which leads to an excessive battery cost and waste of lithium resources.^[^ ^121^ ^]^ However, adopting the strategy of the lithium–carbon hybrid anode can significantly reduce the use of lithium. Ye and co‐workers first proposed to regulate the Li/electrolyte interfacial transfer by plating Li into spherical C granules wired on a 3D conducting skeleton (Figure 6c).^[^ ^36^ ^]^ The spherical carbon consists of highly graphitized carbon layers arranged to form an onion‐like structure with nanogaps in between. After intercalation of Li, an electron deviation from Li to C further elevates the negativity of C atoms, which contributes to a stable Li deposition free of dendrites. More importantly, lithium stored in lithium/carbon compounds significantly improves the CE and achieves an ultralong lifespan of 1000 cycles at a Li surplus as low as 5%. To achieve a longer battery lifespan, the ratio of graphite and lithium needs to be further balanced in the hybrid anode. Jeff Dahn et al. achieved a hybrid anode (890 Wh L^–1^) with an energy density between traditional lithium‐ion batteries and anode‐free lithium metal (Figure 6d).^[^ ^33^ ^]^ By using LiDFOB/LiBF_4_ double salt electrolyte, a constant and fairly high CE for lithium metal cycling (99.6%) was maintained. The high energy of lithium metal can be dispersed among hundreds of conventional lithium‐ion cycles where only graphite is utilized. Moreover, they found that the decay of metal lithium capacity has little to do with the number of cycles completed by graphite capacity. Recently, Zhang et al. proposed a successive conversion−deintercalation (CTD) delithiation mechanism, that is proposed by manipulating the overpotential of the anode to restrain the generation of dead Li.^[^ ^122^ ^]^ In this study, a 1 Ah pouch cell with a CTD delithiation mechanism operates for 150 cycles.

Before solving problems of high‐energy anodes such as silicon and Li metal anode, hybrid anode is a transitional strategy with great commercial application prospects. Through a reasonable structural design, hybrid anode, which is composed of the graphite anode (with long cycle stability and small volume change) and a high specific energy silicon or Li metal anode, can give full play to the advantages of each component. However, there are some problems that hinder its commercialization remaining to be further studied. For instance, how to improve the interface contacts in the hybrid structure, accelerate the conduction of ions and electrons inside the electrode, and relief the stress caused by the volume expansion of silicon or lithium? What's more, the development of electrolyte components that match the hybrid anode will also play a significant role in commercial applications.

## 4 Summary and Outlook

The increasing development of battery‐powered vehicles for exceeding 500 km endurance has stimulated the exploration of lithium batteries with high‐energy‐density and high‐power‐density. In this review, we have screened proximate developments in various types of high specific energy lithium batteries, focusing on silicon‐based anode, phosphorus‐based anode, lithium metal anode, and hybrid anode systems. Among them, silicon‐based anodes and phosphorus‐based anodes have the advantages of high theoretical capacity, environmental friendliness, and abundant reserves. However, both of them have issues like extremely large volume expansion during the lithium insertion process and poor conductivity, which restrict their further practical application. To address these problems, people have proposed strategies based on nanostructure design. Reducing the micrometer‐sized silicon and phosphorus particles to nanoscale is effective in improving its reversibility, electrochemical activity, and structural stability, but the increase in specific surface area for the nanostructured silicon anodes may pose an uncontrollable reaction. Also, there exist great technical challenges associated with the large‐scale and high‐efficient preparation of high‐quality black phosphorus. Li metal anode is considered the most promising anode for the next‐generation battery. For the issues of lithium dendrites and unstable interfaces, many effective strategies have been proposed (electrolyte additives, 3D nanostructure design, SSE, etc.). How to optimize and integrate these strategies to obtain a battery with excellent comprehensive indicators is worthy of further exploration.

As the high specific energy lithium metal anode cannot be put into practical application soon due to their cycle stability and safety issues, and the ratio of Si in the commercialized silicon anode is still low. Therefore, in the future, not only more new electrode structures, and reasonable electrolyte design will require a lot of work, at the same time, hybrid anode, such as silicon–carbon anode, lithium‐ion/lithium metal anode, etc., will need to be further explored. It is noteworthy that the mass ratio between the two mixed anode materials should be strictly controlled to achieve a balance between high energy density and long lifespan. Moreover, attention should also be paid to the design of electrolyte matching with hybrid anode.

Although lithium‐ion battery anodes have experienced a tremendous success, the requirement of higher energy and power density to catch up with the development of market demand is still ongoing. In this process, many issues need in‐depth consideration:

1. SEI is the main factor affecting the stability of electrode interface. The fundamental understanding of the SEI formation mechanism, structure, and component is still inadequate. The insights into the exact role of SEI during cycling should be strongly considered.
2. The solid‐state electrolyte has a significant effect in improving the safety of battery. How to improve the room temperature ionic conductivity and optimize the solid–solid contact need further research.
3. As a scheme to balance high energy density and long cycle stability at present, more work on hybrid anode is needed as a transition strategy before the practical application of high energy density anodes like Li metal anode and Silicon‐based anode.
4. More attention should be paid to the performance of battery in practical applications such as wide working temperature range, and less electrolyte addition (<3 g Ah^–1^).
5. To clearly reveal the reaction mechanism by the in situ or operando techniques in combination with theoretical calculation will be of great importance to help design electrode materials, especially in pouch cells.
6. In special application scenarios, the requirements for flexible, ultrathin, stretchable, or wide temperature range, the corresponding lithium‐ion batteries also need to be explored.

We live in an era of rapid development in the battery field. High specific energy and safe batteries are facing urgent demand in many fields, especially in the field of new energy vehicles, batteries are the biggest bottleneck. With the above possible solutions to further improving core indicators such as specific energy, rate performance, and safety, lithium‐ion batteries are quite promising to be practically applied.

## Conflict of Interest

The authors declare no conflict of interest.
